# Predictive Factors for Sustained Pain after (sub)acute Osteoporotic Vertebral Fractures. Combined Results from the VERTOS II and VERTOS IV Trial

**DOI:** 10.1007/s00270-022-03170-7

**Published:** 2022-06-09

**Authors:** Cristina E. Firanescu, Alexander Venmans, Jolanda de Vries, Paul Lodder, Marinus C. Schoemaker, Albert J. Smeets, Esther Donga, Job R. Juttmann, Karen Schonenberg, Caroline A. H. Klazen, Otto E. H. Elgersma, Frits H. Jansen, Hendrik Fransen, Joshua A. Hirsch, Paul N. M. Lohle

**Affiliations:** 1 Department of Radiology, Elisabeth TweeSteden Hospital, Hilvarenbeekseweg 60, 5022 GC Tilburg, Netherlands; 2 Department of Medical Psychology, Elisabeth TweeSteden Hospital, Hilvarenbeekseweg 60, 5022 GC Tilburg, Netherlands; 3 Department of Medical and Clinical Psychology, Tilburg University, Warandelaan 2, 5037 AB Tilburg, Netherlands; 4 Department of Radiology, Medisch Spectrum Twente, Koningsplein 1, 7512 KZ Enschede, Netherlands; 5 Department of Radiology, Albert Schweitzer Hospital, Albert Schweitzerplaats 25, 3318 AT Dordrecht, Netherlands; 6 Department of Radiology, Catharina Hospital, Michelangelolaan 2, 5623 EJ Eindhoven, Netherlands; 7 Department of Radiology, AZ St Lucas, Groenebril 1, 9000 Ghent, Belgium; 8 Department of Radiology, Massachusetts General Hospital, Boston, MA USA; 9 Present Address: Department of Radiology, VieCuri Hospital, Tegelseweg 21, 5912 BL Venlo, Netherlands

**Keywords:** Percutaneous vertebroplasty (PV), Osteoporotic vertebral compression fracture (OVCF), Visual analogue scale (VAS), Polymethylmethacrylate (PMMA)

## Abstract

**Purpose:**

Osteoporotic vertebral compression fractures are treated conservatively or in selected cases with percutaneous vertebroplasty (PV). The purpose of this retrospective analysis is to determine predictive factors for a high visual analogue scale (VAS) pain score after conservative, sham or PV and is based on previously published randomized trials.

**Methods:**

The VERTOS II compared conservative versus PV, and VERTOS IV compared sham versus PV treatment. The conservative group received pain medication. The sham and PV group received subcutaneous lidocaine/bupivacaine. In addition, the PV group received cementation, which was simulated in the sham group. Nineteen different predictors of high (≥ 5) versus low (< 5) VAS pain score at 12 months were investigated.

**Results:**

20.7% of patients in the PV group demonstrated a VAS ≥ 5 at the 12-month, compared to 40.1% in the conservative or sham group, with a significant difference (*χ*^2^(1) = 15.26, *p* < 0.0001, OR = 2.57, 95% CI = 1.59 to 4.15). In the subgroup analysis, we detected five predictors for the risk of high pain scores (VAS ≥ 5 after 12 months follow-up), namely: female, baseline VAS > 8, long-term baseline pain, mild/severe Genant and new fractures.

**Conclusions:**

Statistically significant more patients had a high pain score at 12 months in the sham and conservative group when compared with the PV group. Five predictors were identified for sustained high local back pain, regardless of the received treatment. Patients with moderate fracture deformity were less likely to have high pain scores at 12 months if they received PV than if they had sham or conservative therapy.

**Supplementary Information:**

The online version contains supplementary material available at 10.1007/s00270-022-03170-7.

## Introduction

Osteoporotic vertebral compression fractures (OVCFs) are commonly encountered in the elderly population. In Europe, the incidence of a new OVCF is estimated at age 75–79 years as 2.9% per year in women and 1.4% per year in men [1]. OVCFs are associated with kyphotic deformity, back pain and consequently a reduction in quality of life [2]. When conservative therapy leads to insufficient pain relief, percutaneous vertebroplasty (PV) can be considered as a treatment option. PV is a minimally invasive image-guided procedure involving injection of polymethylmethacrylate (PMMA) into a fractured vertebral body, aiming to provide pain relief and stability. This technique became initially widespread based on preliminary observational trial results [3, 4].

In 2010, VERTOS II (an open-label randomized controlled trial) demonstrated that PV resulted in pain reduction, more rapid pain relief, and improved quality of life [5]. VERTOS II provisionally enrolled patients with pain duration up to 6-weeks which extended up to 9 weeks or more at time of PV, and compared conservative treatment with PV. VERTOS IV included provisionally enrolled patients with pain duration up to 9 weeks which seems to have extended to 12 weeks or more at the time of PV. PV occurred significantly later than provisional enrolment in both trials. The VERTOS IV trial aimed to clarify the role of PV using a double-blind randomized controlled trial with a sham control [6]. This trial showed no significant differences in pain relief at 12 months between the PV group and the sham group for (sub)acute OVCFs. In the VERTOS IV double-blind randomized control trial, we discovered that within the group of patients with persisting high pain levels at 12 months follow-up, considerable more patients were in the sham group compared to the PV group. In this article, we elaborate more on this observation.

To examine this observation, we combined the databases from the VERTOS II and VERTOS IV trial, providing data from patients who were treated conservatively, with sham, or with a PV intervention.

Both trials specifically looked at (sub)acute OVCFs. The inclusion criteria, the study design and PV technique were the same in both trials. The aim of this article is to determine predictive factors for a high VAS pain score after conservative, sham or PV based on the results of these two RCTs.

## Method

### Study Design

Both VERTOS II and VERTOS IV were multi-centre randomized trials: one an open-label and the other a blinded trial. Both trials were approved by the Institutional review board as open-label and multi-centre randomized trials performed in community hospitals in the Netherlands and Belgium. The studies obtained ethics approval of all participating hospitals. All patients gave written informed consent before taking part in one of the trials. Both trials were registered at ClinicalTrials.com (VERTOS II NCT00232466 and VERTOS IV NCT01200277). The detailed description of the trials methods can be found online. [5, 6]

### Inclusion criteria

Inclusion criteria were the same in both trials: age 50 years or more, 1–3 vertebral OVCFs with 15% or more loss of vertebral body height, bone oedema on MRI, T5-L5 focal back pain at the level of fracture, score of 5 or higher on a VAS, and decreased bone density (T-score less than -1). The fracture duration at time of provisional enrolment extended to 6-weeks in VERTOS II and 9-weeks in VERTOS IV, due to slow recruitment.

In both trials, all patients were referred by the general practitioner for spinal radiography. Patients with radiographic evidence of fracture were administered a questionnaire. Those patients found to have a crush fracture and reporting pain ≥ 5/10 and pain duration less than 6 weeks (VERTOS II) and 9 weeks (VERTOS IV) were provisionally enrolled pending physician consult and MRI to confirm eligibility. PV occurred later—with a mean delay of 9 days in VERTOS II and a median of 11 days (IQR 7–18 days) in the VERTOS IV trial.

Both trials had similar exclusion criteria and included outpatients only.

In the VERTOS II trial, the participants were randomly allocated to conservative treatment or PV, i.e. subcutaneous and periosteal infiltration with 1% lidocaine (lignocaine) followed by injection of PMMA under fluoroscopy guidance. In the VERTOS IV trial the participants received local infiltration with 1% lidocaine (lignocaine) and 0.25% bupivacaine, bone biopsy needles against the pedicles and then PV or a sham procedure. The PV procedure consisted of injection of PMMA under fluoroscopic guidance.

### Participant Flow and Recruitment

In VERTOS II trial, 202 patients were recruited and analysed between 2005 and 2008 and randomly allocated to PV (101 patients) or conservative treatment (101 patients). In VERTOS IV trial, 180 patients were recruited and analysed between 2011 and 2014 and randomly allocated to PV (90 patients) or sham procedure (86 patients). The flow diagrams explaining the enrolments and outcomes from the both trials are shown and can be assessed in the original articles. [5, 6]

### Outcome Measures

In both trials, VAS ≥ 5 was the main inclusion criterion and considered as a high pain score. Therefore, in the current paper the identical threshold for a high pain score was used, but at 12-monthfollow-up. The analysed groups are: the conservative group (from VERTOS II), the sham group lidocaine/ bupivacaine (from VERTOS IV group) and the PV group (from VERTOS II and IV). The assessed risk factors considered to play a possible role in sustained pain (VAS ≥ 5) at 12-month follow-up were the baseline characteristics: age, gender, days of pain (defined as number of days between pain onset and treatment), a high VAS at baseline (≥ 8), number of fractures at baseline, number of treated fractures, level of treated fractures (Th1-Th10, Th11-L2, L3-L5), type of fracture assessed according to the Genant classification (mild, moderate or severe, wedge or biconcave)[7], T-score graded as low (between 0 to − 2.5), medium (− 2.5 to − 4) and high (− 4 to − 6) [8] and the follow-up characteristics, further vertebral height loss and new fractures during follow-up.

### Statistics

After merging the VERTOS II and IV datasets, baseline characteristics were calculated separately for the PV, sham and conservative groups. Frequencies and percentages were calculated for categorical baseline characteristics, means and standard deviations for normally distributed characteristics, and the median and interquartile range for non-normally distributed characteristics. The percentage of patients with VAS scores of 5 or higher at the 12-month follow-up were determined for each of the three treatments. A Chi-square test was used to test the null hypothesis that these percentages were independent of treatment. For each treatment and study, logistic regression analyses were used to assess what factors predict patients having a VAS score of 5 or higher at the 12-month follow-up. The resulting estimates were shown in forest plots using the R package metafor (version 2.0). The logistic regression analyses were conducted using the free software R, and all other analyses were conducted using IBM SPSS (version 25). Odds ratios and 95% confidence intervals were computed as effect size in the logistic regression analyses. *P*-values smaller than 0.05 were considered statistically significant. In the subgroup analyses, a Bonferroni correction was applied to adjust the significance level for multiple testing.

## Results

### Baseline Characteristics

The baseline characteristics were similar in the three groups, respectively, PV patients from the VERTOS II and VERTOS IV, conservative-treated patients from the VERTOS II and sham patients from the VERTOS IV trial. Both VERTOS II and IV included patients with VAS ≥ 5 at baseline. The average VAS score at baseline was 7.74 (SD = 1.51). The range of fracture duration extended up to 92 days in the VERTOS II trial according to trial manuscript. The median duration of back pain at the time of PV in VERTOS IV was reported as 43 days, which indicates that half the patients had acute fractures (< 6/52 weeks) and half had had sub-acute fractures (6–12/52 weeks) at the time of PV.

The difference in baseline characteristics, in terms of back pain duration in days before treatment, between conservative treatment versus sham and PV, can be explained by the fact that conservative treatment was the only treatment in which analgesics were given immediately following randomization during outpatient visits, whereas PV took place a few days later. Randomization in VERTOS IV took place in the angio room a few days after outpatient visit (Table 1).

**Table 1:** Summary of baseline characteristics

	PV (N = 101 + 90 = 191)^**#**^	SHAM (*N* = 86)	Conservative (*N* = 101)
Mean (SD) age (years)	75.1 (10.1)	76.9 (8.1)	75.3 (8.5)
Women	136 (72%)	66 (77%)	69 (68%)
Median (interquartile range) days local back pain before procedure	38 (25–53)	36 (25–51)	25 (14–37)
Number of OVCFs at baseline	251	108	120
Type of fracture (Genant classification)*			
Mild (10–20%)	94 (37%)	30 (28%)	55 (46%)
Moderate (20–40%)	109 (43%)	49 (45%)	45 (38%)
Severe (> 40%)	48 (19%)	30 (28%)	20 (17%)
Wedge	146 (58%)	65 (60%)	97 (81%)
Biconcave	105 (42%)	44 (40%)	23 (19%)
Crush	0 (0%)	0 (0%)	0 (0%)
*Vertebral level with bone oedema*			
Th5-Th10	55 (22%)	24 (22%)	32 (25%)
Th11-L2	150 (59%)	69 (64%)	66 (52%)
L3-L5	49 (19%)	15 (14%)	28 (22%)
*No. of spinal levels treated*			
1	140 (74%)	66 (61%)	70 (74%)
2	36 (19%)	15 (28%)	17 (18%)
3 or more	13 (7%)	4 (11%)	7 (8%)
Bone density T-score	-2.7 (1.1)	-2.4 (0.9)	-3.0 (1.0)
Drugs for osteoporosis	66 (35%)	49 (57%)	26 (26%)
Initial pain treatment	182 (95%)	78 (91%)	94 (93%)
Initial VAS score	7.8 (1.4)	7.9 (1.6)	7.5 (1.6)

^**#**^101 patients from VERTOS II and 90 patients from VERTOS IV trial

^*^Indicates percentage loss of vertebral body height

### VAS Scores 5 or Higher at 12 months

Table 2 shows separately for each study and treatment the percentage of patients with VAS ≥ 5 at the 12-month follow-up. In VERTOS II, 30.3% of all patients showed a VAS ≥ 5 at 12 months, comparable to 30.1% of all patients in VERTOS IV, a non-significant difference (*χ*^2^(1) = 0.003, *p* = 0.960). In VERTOS II, 21.3% of the patients in the PV group showed a VAS ≥ 5, as opposed to 39.4% of the patients who received conservative treatment, with a significant difference (*χ*^2^(1) = 7.28, *p* = 0.007, OR = 2.40, 95% CI = 1.26 to 4.57). Similarly, in VERTOS IV, 18% of the patients in the PV group showed a VAS ≥ 5 at the 12-month follow-up, compared to 41.1% of the patients in the sham group, with a significant difference (*χ*^2^(1) = 8.08, *p* = 0.005, OR = 2.79, 95% CI = 1.36 to 5.73). When combining the data of VERTOS II and IV, 20.7% of the patients in the PV group reported a VAS ≥ 5 at the 12-month follow-up, compared to 40.1% of the patients receiving no PV treatment (conservative or sham), with a significant difference (*χ*^2^(1) = 15.26, *p* < 0.0001, OR = 2.57, 95% CI = 1.59 to 4.15).

**Table 2:** For each study and treatment, the number (%) of patients with VAS ≥ 5 at 12-month follow-up

VERTOS	PV	Conservative	SHAM	No PV	Total
II	20 (21.3%)	37 (39.4%)	–	37 (39.4%)	57 (30.3%)
IV	16 (18.0%)	–	30 (41.1%)	30 (41.1%)	46 (30.1%)
II + IV	36 (20.7%)	37 (39.4%)	30 (41.1%)	67 (40.1%)	103 (30.2%)

**Fig. 1:**
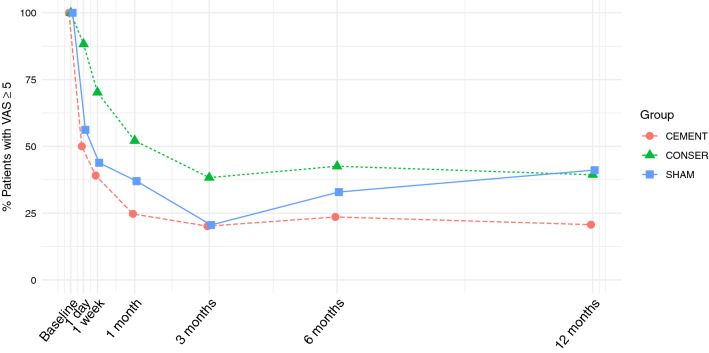
The percentage of patients with VAS ≥ 5 during the 12-month follow-up outlined for each type of treatment

Figure 1 illustrates for each treatment the percentage of patients with VAS ≥ 5 during the 12 month follow-up of pain scores at the 12-month follow-up.

### Predictors of High VAS Scores at 12 months

Patients in the PV groups of the VERTOS II and VERTOS IV trials demonstrated significantly less often VAS ≥ 5 at 12 months follow-up compared to the sham and conservative groups. We analysed the characteristics of patients having a VAS ≥ 5 at 12 months.

**Fig. 2:**
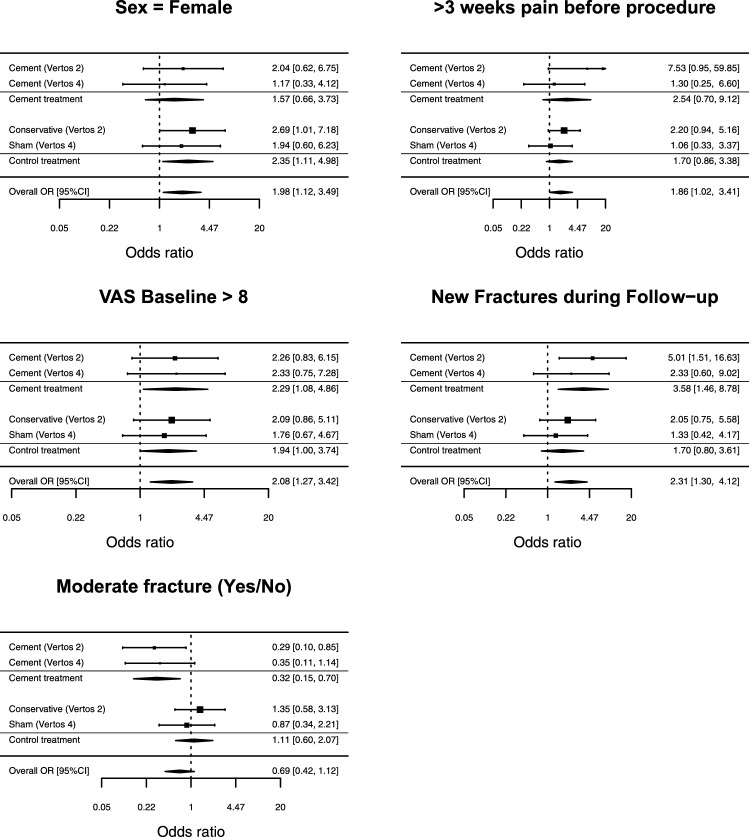
Forest plots showing for each study and treatment the effect of five predictors on having VAS ≥ 5 at 12-month follow-up

Figure 2 illustrates forest plots for five significant predictors of VAS ≥ 5 at 12 months. In each forest plot, the estimated odds ratio and 95% confidence interval are shown separately for each study and treatment. The odds ratios are also pooled across PV and non-PV treatments, as well as pooled across all treatments. Confidence intervals not containing one indicate a statistically significant odds ratio.

Five characteristics were predictive of VAS ≥ 5 at the 12-month follow-up. Females demonstrated higher odds than males of having a VAS ≥ 5 at the 12-month follow-up (OR = 1.98, 95%CI = 1.12 to 3.49). Patients who were in pain > 3 weeks before the intervention showed a higher odds of having a VAS ≥ 5 at 12-month follow-up than patients who experienced pain for a shorter time duration (OR = 1.86, 95%CI = 1.02 to 3.41). Patients with a baseline VAS > 8 showed higher odds of having a VAS ≥ 5 at 12-month follow-up compared to those with lower baseline VAS scores (OR = 2.08, 95%CI = 1.27 to 3.42). Patients with new OVCFs during the follow-up period showed a higher odds on VAS ≥ 5 at 12 months than patients without new fractures during follow-up (OR = 2.31, 95%CI = 1.30 to 4.12). These four effects were pooled across all treatments, because the difference between treatments in these effects did not reach statistical significance.

In each dataset and treatment, patients of older age appeared to have a higher odds of having a VAS ≥ 5 at 12-month follow-up. However, these results failed to reach statistical significance (OR = 1.51, 95%CI = 0.93 to 2.45). Supplemental Figure S1 presents the odds ratio effects for predictors that were not significantly associated with a VAS ≥ 5 at 12-month follow-up.

Patients with a moderate vertebral body fracture showed a lower odds on having a VAS ≥ 5 after PV, than patients with no moderate fracture classification (OR = 0.32, 95%CI = 0.15 to 0.70). This effect was not found for either of the control treatments (OR = 1.11, 95%CI = 0.60 to 2.07), and the difference between these effects in the two conditions was statistically significant (*Z* = 2.45, *p* = 0.014).

## Discussion

The results of VERTOS II and VERTOS IV showed that at 12-monthfollow-up patients from the conservative and sham group had more often high pain scores (VAS ≥ 5) than patients who underwent PV. There are four predictors found for the risk of high pain scores (VAS ≥ 5) after 12-monthfollow-up, namely female, baseline VAS > 8, pain > 3 weeks before procedure and new OVCFs. When these four predictors hold true, there is increased risk of high VAS after 12-month follow-up, with no significant pain outcome difference between conservative therapy, sham or PV (no policy difference). However, moderate vertebral body height loss was significantly more predictive of low pain scores at 12 months after PV than after other treatments.

OVCFs can cause severe back pain and kyphosis with a negative impact on morbidity and quality of life. The retrospective and sham trials conducted until present showed contradictory results [5, 6, 9–11]. Consequently, various and different recommendations are presented for patient selection and optimal treatment strategies for painful OVCFs [12, 13].

In daily practice, clinicians taking care of OVCF patients are faced with the dilemma of whether to treat an individual patient with augmentation or conservative therapy. This paper analysed data and identified factors from two previous published RCTs (VERTOS II and VERTOS IV) that can influence the clinical outcome (pain relief) at 12 months. This may help to identify the patients who most likely benefit from PV.

From the combined results of the two VERTOS trials, we found that significantly more patients in the sham and conservative group had a high pain score (VAS ≥ 5) at 12-month follow-up than those in the PV group (40.1 vs 20.7%). When investigating the demographic and clinical characteristics, we identified 5 predictors for sustained high local back pain: female gender, patients with a baseline VAS > 8, pain duration > 3 weeks until treatment, mild or severe fracture classification and new fractures during follow-up. Female gender is more predisposed to osteoporosis and subsequent new fractures. Severe fractures causing kyphosis, heal slower and probably are much more painful. These factors probably combine and result in a high pain score after 12 months. Neither the number of treated levels nor the fracture level were significant predictors of high VAS scores at 12 months.

Odds ratio effect sizes of two dichotomous predictors can be interpreted inversely [14]. Therefore, our results indicate that four predictors [male, < 3 weeks of pain before treatment, baseline 5 < VAS < 8, without new fractures during follow-up] provide higher probability of successful pain reduction at 12 months after treatment of (sub)acute OVCFs (both in conservative, sham and PV). In the case of moderate (sub)acute OVCF, there is also a higher probability of successful pain reduction, but *only* after PV and not after conservative or sham intervention. The practical implication is that patients with moderate (sub)acute OVCFs are particularly suited to PV and not conservative treatment or sham intervention. Although no definitive recommendation can be made for an individual patient based on these findings, the combined results of these two trials provide additional perspective and further nuance to the VERTOS IV conclusion.

In contrast to VERTOS II, VERTOS IV on average did not indicate any added value of PV regarding pain relief compared to sham. However, it may be important to identify individual characteristics of patients for whom PV does result in significant pain reduction. Indeed, we must realize that there seems to be a distinct subgroup of patients with (sub)acute OVCFs and certain baseline characteristics predictive of successful pain relief after PV as opposed to the general conclusion of VERTOS IV.

The VAPOUR trial showed clinical success for PV over placebo for patients meeting the inclusion criteria severe pain (VAS ≥ 7) at baseline, OVCFs of less than 6 weeks duration and OVCFs at the thoracolumbar spinal segment as predictors for clinical success [11]. Although these results suggest that patients with severe pain and younger fractures may benefit from a PV intervention, the current study provides a more nuanced perspective. First, younger fracture age was predictive of low pain at 12 months, yet this effect was found regardless of the received intervention. Second, we found that patients with severe pain (VAS > 8) at baseline showed a higher chance on having VAS of 5 or higher at 12 months, regardless of the received intervention. Although VAPOUR showed on average a clinical success for PV compared to sham in a population of patients with severe pain (VAS ≥ 7), the study did not investigate the characteristics of patients who benefited most from PV treatment. The currently study revealed that patients with a moderate vertebral fracture type benefit most from PV. Identification of such characteristics may contribute to better select patients with OVCFs for PV. This approach seems a rational step, balancing between the risk of overtreating or undertreating this fragile population.

Along with the worldwide discussion on PV, this paper discusses the selection of patients for PV. VERTOS IV was the 3rd negative placebo trial of PV in contrast to the VAPOUR trial [11], which was the only positive placebo trial for PV, and was contemporary with VERTOS IV. Among other differences, the key differences in patient selection between VERTOS IV and VAPOUR are (1) vertebral fracture duration at the time of PV, (2) pain severity at baseline and (3) inclusion of inpatients. Mean fracture duration in VAPOUR was 2.6 weeks compared to 6.1 weeks in VERTOS IV, and this may be the critical factor explaining the different outcomes in these two trials. The pain severity at baseline was 8.6 on VAS for VAPOUR whereas 7.8 for VERTOS IV. In VAPOUR, 59% of patients were hospitalized in contrast to 0% inpatients in VERTOS IV. These trial differences and more information are described in the publication by Diamond et al.[15]. Compared with the other RCTs, VAPOUR used a different clinical approach offering PV much earlier compared to a longer fracture duration up to 12 months in the Kallmes’ and Buchbinder studies and up to 12 weeks in VERTOS IV. In addition, VAPOUR used a different cementation technique to the trials by Kallmes and Buchbinder. The “vertebral fill” technique is designed to brace the whole vertebral body against instability, augmenting it and preventing further collapse. The mean volume of PMMA injected into the vertebral body was three times greater in VAPOUR (7.5 mL) than in the Kallmes (2.6 mL).

## Limitations

These results are based on a large number of subgroup analyses. Based on chance alone, some significant differences are expected. However, we adjusted our significance level for multiple comparisons, minimizing the risk on false positive conclusions. Because of the exploratory nature of these analyses, the findings should be considered as new hypotheses to test in future research. These results are limited to the cohort studied in VERTOS II and VERTOS IV trials. Hospitalized patients were not included, so the findings may not apply to them. It is not clear how many patients had fracture duration < 3 weeks at the time of PV, so findings may not apply to patients with early fractures.

## Conclusions

The combined treatment results of (sub)acute OVCFs from the VERTOS II and VERTOS IV trials demonstrated that statistically significant more patients had a high pain score at 12 months in the sham and conservative group when compared with the PV group. Five predictors were identified for sustained high local back pain, regardless of the received treatment: new fractures during follow-up, female gender, patients with a baseline VAS score higher than 8, mild or severe fracture classification and more days of pain until treatment. Patients with moderate fracture deformity (on a Genant scale) were less likely to have high pain scores at 12 months if they received PV than if they had sham or conservative therapy.

## Supplementary Information

Below is the link to the electronic supplementary material.

Supplementary file1 (PDF 12 KB)
