# Supplementary material for: Predictive Factors for Sustained Pain after (sub)acute Osteoporotic Vertebral Fractures. Combined Results from the VERTOS II and VERTOS IV Trial
Source: Cardiovasc Intervent Radiol. 2022 Jun 9;45(9):1314–21. doi: 10.1007/s00270-022-03170-7 (PMC9458567; doi:10.1007/s00270-022-03170-7)
Supplement: Supplementary file 1 — Supplementary file1 (PDF 12 KB) [file 270_2022_3170_MOESM1_ESM.pdf]

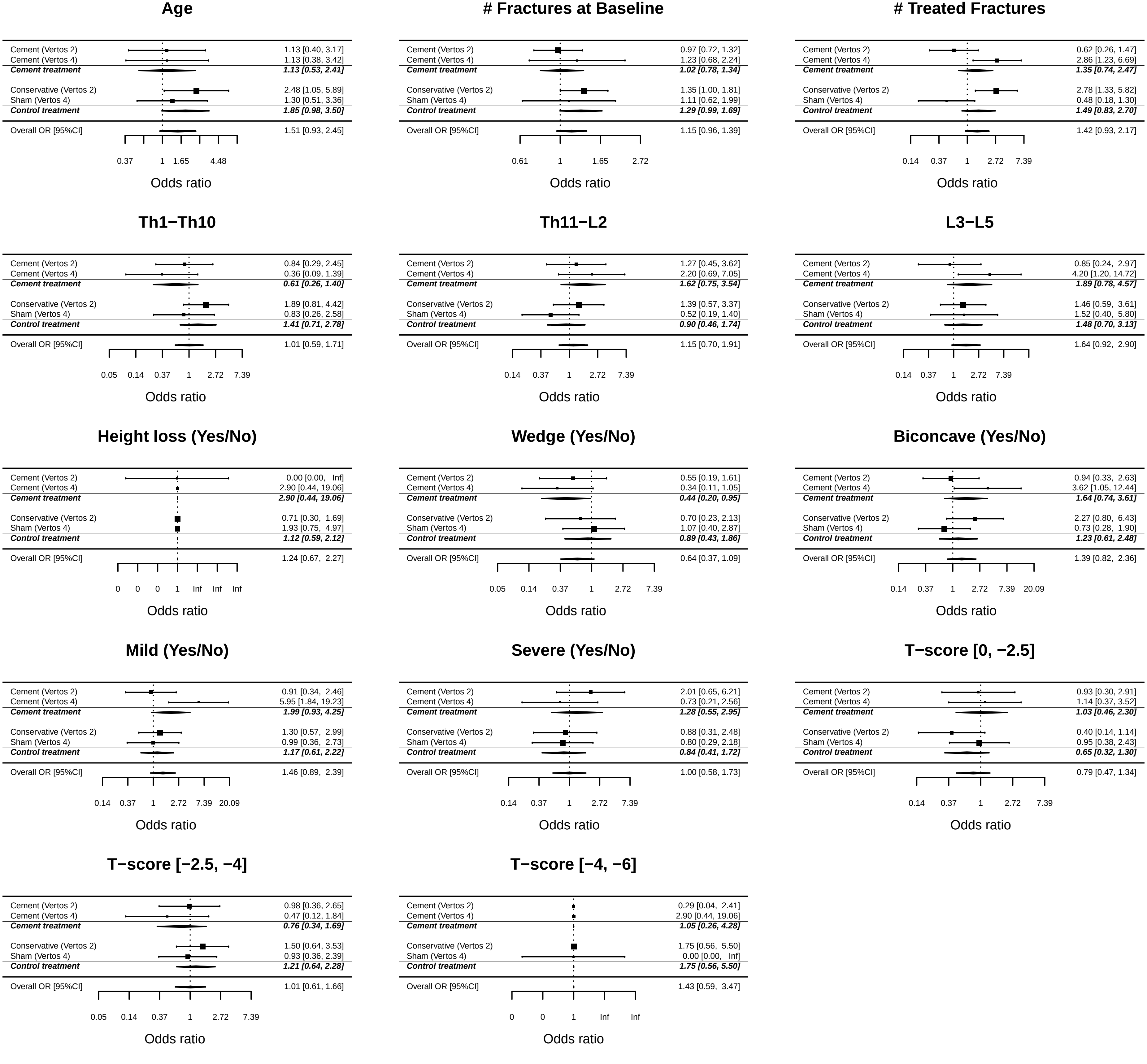

Age
Cement (Vertos 2) 1.13 [0.40, 3.17]
Cement (Vertos 4) 1.13 [0.38, 3.42]
Cement treatment 1.13 [0.53, 2.41]
Conservative (Vertos 2) 2.48 [1.05, 5.89]
Sham (Vertos 4) 1.30 [0.51, 3.36]
Control treatment 1.85 [0.98, 3.50]
Overall OR [95%CI] 1.51 [0.93, 2.45]
0.37 1 1.65 4.48
Odds ratio
Fractures at Baseline
Cement (Vertos 2) 0.97 [0.72, 1.32]
Cement (Vertos 4) 1.23 [0.68, 2.24]
Cement treatment 1.02 [0.78, 1.34]
Conservative (Vertos 2) 1.35 [1.00, 1.81]
Sham (Vertos 4) 1.11 [0.62, 1.99]
Control treatment 1.29 [0.99, 1.69]
Overall OR [95%CI] 1.15 [0.96, 1.39]
0.61 1 1.65 2.72
Odds ratio
Treated Fractures
Cement (Vertos 2) 0.62 [0.26, 1.47]
Cement (Vertos 4) 2.86 [1.23, 6.69]
Cement treatment 1.35 [0.74, 2.47]
Conservative (Vertos 2) 2.78 [1.33, 5.82]
Sham (Vertos 4) 0.48 [0.18, 1.30]
Control treatment 1.49 [0.83, 2.70]
Overall OR [95%CI] 1.42 [0.93, 2.17]
0.14 0.37 1 2.72 7.39
Odds ratio
Th1−Th10
Cement (Vertos 2) 0.84 [0.29, 2.45]
Cement (Vertos 4) 0.36 [0.09, 1.39]
Cement treatment 0.61 [0.26, 1.40]
Conservative (Vertos 2) 1.89 [0.81, 4.42]
Sham (Vertos 4) 0.83 [0.26, 2.58]
Control treatment 1.41 [0.71, 2.78]
Overall OR [95%CI] 1.01 [0.59, 1.71]
0.05 0.14 0.37 1 2.72 7.39
Odds ratio
Th11−L2
Cement (Vertos 2) 1.27 [0.45, 3.62]
Cement (Vertos 4) 2.20 [0.69, 7.05]
Cement treatment 1.62 [0.75, 3.54]
Conservative (Vertos 2) 1.39 [0.57, 3.37]
Sham (Vertos 4) 0.52 [0.19, 1.40]
Control treatment 0.90 [0.46, 1.74]
Overall OR [95%CI] 1.15 [0.70, 1.91]
0.14 0.37 1 2.72 7.39
Odds ratio
L3−L5
Cement (Vertos 2) 0.85 [0.24, 2.97]
Cement (Vertos 4) 4.20 [1.20, 14.72]
Cement treatment 1.89 [0.78, 4.57]
Conservative (Vertos 2) 1.46 [0.59, 3.61]
Sham (Vertos 4) 1.52 [0.40, 5.80]
Control treatment 1.48 [0.70, 3.13]
Overall OR [95%CI] 1.64 [0.92, 2.90]
0.14 0.37 1 2.72 7.39
Odds ratio
Height loss (Yes/No)
Cement (Vertos 2) 0.00 [0.00, Inf]
Cement (Vertos 4) 2.90 [0.44, 19.06]
Cement treatment 2.90 [0.44, 19.06]
Conservative (Vertos 2) 0.71 [0.30, 1.69]
Sham (Vertos 4) 1.93 [0.75, 4.97]
Control treatment 1.12 [0.59, 2.12]
Overall OR [95%CI] 1.24 [0.67, 2.27]
0 0 0 1 Inf Inf Inf
Odds ratio
Wedge (Yes/No)
Cement (Vertos 2) 0.55 [0.19, 1.61]
Cement (Vertos 4) 0.34 [0.11, 1.05]
Cement treatment 0.44 [0.20, 0.95]
Conservative (Vertos 2) 0.70 [0.23, 2.13]
Sham (Vertos 4) 1.07 [0.40, 2.87]
Control treatment 0.89 [0.43, 1.86]
Overall OR [95%CI] 0.64 [0.37, 1.09]
0.05 0.14 0.37 1 2.72 7.39
Odds ratio
Biconcave (Yes/No)
Cement (Vertos 2) 0.94 [0.33, 2.63]
Cement (Vertos 4) 3.62 [1.05, 12.44]
Cement treatment 1.64 [0.74, 3.61]
Conservative (Vertos 2) 2.27 [0.80, 6.43]
Sham (Vertos 4) 0.73 [0.28, 1.90]
Control treatment 1.23 [0.61, 2.48]
Overall OR [95%CI] 1.39 [0.82, 2.36]
0.14 0.37 1 2.72 7.39 20.09
Odds ratio
Mild (Yes/No)
Cement (Vertos 2) 0.91 [0.34, 2.46]
Cement (Vertos 4) 5.95 [1.84, 19.23]
Cement treatment 1.99 [0.93, 4.25]
Conservative (Vertos 2) 1.30 [0.57, 2.99]
Sham (Vertos 4) 0.99 [0.36, 2.73]
Control treatment 1.17 [0.61, 2.22]
Overall OR [95%CI] 1.46 [0.89, 2.39]
0.14 0.37 1 2.72 7.39 20.09
Odds ratio
Severe (Yes/No)
Cement (Vertos 2) 2.01 [0.65, 6.21]
Cement (Vertos 4) 0.73 [0.21, 2.56]
Cement treatment 1.28 [0.55, 2.95]
Conservative (Vertos 2) 0.88 [0.31, 2.48]
Sham (Vertos 4) 0.80 [0.29, 2.18]
Control treatment 0.84 [0.41, 1.72]
Overall OR [95%CI] 1.00 [0.58, 1.73]
0.14 0.37 1 2.72 7.39
Odds ratio
T−score [0, −2.5]
Cement (Vertos 2) 0.93 [0.30, 2.91]
Cement (Vertos 4) 1.14 [0.37, 3.52]
Cement treatment 1.03 [0.46, 2.30]
Conservative (Vertos 2) 0.40 [0.14, 1.14]
Sham (Vertos 4) 0.95 [0.38, 2.43]
Control treatment 0.65 [0.32, 1.30]
Overall OR [95%CI] 0.79 [0.47, 1.34]
0.14 0.37 1 2.72 7.39
Odds ratio
T−score [−2.5, −4]
Cement (Vertos 2) 0.98 [0.36, 2.65]
Cement (Vertos 4) 0.47 [0.12, 1.84]
Cement treatment 0.76 [0.34, 1.69]
Conservative (Vertos 2) 1.50 [0.64, 3.53]
Sham (Vertos 4) 0.93 [0.36, 2.39]
Control treatment 1.21 [0.64, 2.28]
Overall OR [95%CI] 1.01 [0.61, 1.66]
0.05 0.14 0.37 1 2.72 7.39
Odds ratio
T−score [−4, −6]
Cement (Vertos 2) 0.29 [0.04, 2.41]
Cement (Vertos 4) 2.90 [0.44, 19.06]
Cement treatment 1.05 [0.26, 4.28]
Conservative (Vertos 2) 1.75 [0.56, 5.50]
Sham (Vertos 4) 0.00 [0.00, Inf]
Control treatment 1.75 [0.56, 5.50]
Overall OR [95%CI] 1.43 [0.59, 3.47]
0 0 1 Inf Inf
Odds ratio
